# A Clearing Technique to Enhance Endogenous Fluorophores in Skin and Soft Tissue

**DOI:** 10.1038/s41598-019-50359-x

**Published:** 2019-10-31

**Authors:** Deshka S. Foster, Alan T. Nguyen, Malini Chinta, Ankit Salhotra, R. Ellen Jones, Shamik Mascharak, Ashley L. Titan, R. Chase Ransom, Oscar L. da Silva, Eliza Foley, Emma Briger, Michael T. Longaker

**Affiliations:** 1 Hagey Laboratory for Pediatric Regenerative Medicine, Stanford University School of Medicine, Stanford, CA 94305 USA; 2 Department of Surgery, Stanford University School of Medicine, Stanford, CA 94305 USA

**Keywords:** Microscopy, Stem cells

## Abstract

Fluorescent proteins are used extensively in transgenic animal models to label and study specific cell and tissue types. Expression of these proteins can be imaged and analyzed using fluorescent and confocal microscopy. Conventional confocal microscopes cannot penetrate through tissue more than 4–6 μm thick. Tissue clearing procedures overcome this challenge by rendering thick specimens into translucent tissue. However, most tissue clearing techniques do not satisfactorily preserve expression of endogenous fluorophores. Using simple adjustments to the BABB (Benzoic Acid Benzyl Benzoate) clearing methodology, preservation of fluorophore expression can be maintained. Modified BABB tissue clearing is a reliable technique to clear skin and soft tissue specimens for the study of dermal biology, wound healing and fibrotic pathologies.

## Introduction

Fluorescent proteins are widely used in transgenic animals to allow for the visualization and isolation of specific cell and tissue types. Endogenous fluorophore expression in transgenic animal models permits lineage tracing of specific cell types, facilitating the study of many disease types and therapeutics *in vivo*^1^. The Cre/lox recombination system is often used to drive expression of such proteins. Cre/lox activity can be spontaneous or made conditional by linking with the ligand-binding domain of the estrogen receptor (Cre-ER^T2^) for example, which then requires Tamoxifen administration to induce expression. Antibiotic and viral constructs are used to achieve similar results^2^. Examples of endogenous fluorophore mouse models frequently used with the Cre-lox system include Rosa^mTmG^ and the Rainbow mouse^3–5^.

Technology available to visualize and image tissues expressing fluorescent proteins continues to expand^6^. In particular, confocal microscopy (laser confocal scanning microscopy) provides highly detailed characterization of cellular and tissue biology by utilizing laser power to excite fluorophores and scanning mirrors to remove blur^7^. Sectioned tissue is commonly used because confocal microscopy can generally penetrate these thin slices to effectively capture fluorophore expression. However, sectioned tissue presents several limitations, including that it may provide a limited view of the biology given that not all cell/tissue relationships are maintained depending upon the dimension in which the tissue is sectioned. Critical pathologic or biologic areas of interest may be lost secondary to trimming of tissue specimens. Additionally, cryosectioning requires significant time. If reconstruction of contiguous tissue architecture is necessary for the research aims, specialized software and skill is required to recreate the specimen macro-structure^8,9^.

Whole-mounting is an alternative to tissue sections and is useful for the study of intact organs and tissue specimens, permitting a comprehensive view of the three-dimensional biology. For example, confocal microscopy imaging of intact brain tissue whole-mounts allows visualization and mapping of complete neuronal networks^10^. Another benefit of whole-mounting is that the tissue remains intact, removing the possibility for potential loss of crucial areas of a given sample^11^. However, a challenge of imaging whole mount tissue remains that confocal microscopes are generally unable to penetrate through samples more than 4–6 μm thick. This is related to uneven and varied refraction indexes in biological tissue. For example, interstitial fluid contains a low refractive index while fatty material in cell membranes contains a high refractive index of about 1.45^12^. Dense connective tissues, such as those seen in wound healing and fibrotic samples, will have different refractive indexes compared with healthy surrounding tissues. As a result of this variation, the three-dimensional visualization capability of confocal microscopy has not yet been fully realized^12^.

Tissue clearing techniques improve the penetration of microscopy by targeting lipids to maintain a stable refraction index throughout a tissue sample. This is achieved by dehydrating the tissue and solvating the lipids. The dehydration process adjusts the refraction index of most proteins to be above 1.5^12^, while lipid solvation causes the tissue to become translucent. With evenly matched refraction indexes, tissue clarity increases allowing microscopic imaging of thicker tissue specimens^12^. Additionally, use of a mounting media with a similar refractive index to the cleared tissue, can further improve the clarity. A variety of methods exist to clear tissue samples, including both organic solvent-based and aqueous-based protocols^12^, including BABB (Benzoic Acid| Benzyl Benzoate), 3DISCO (Three-Dimensional Imaging of Solvent-Cleared Organs), DBE (DiBenzyl Ether), ScaleA2 (a combination of urea, glycerol, and Triton X-100), CUBIC (Clear, Unobstructed Brain Imaging Cocktails and computational analysis), CLARITY (Clear Lipid-exchanged Acrylamide-hybridized Rigid Imaging/Immunostaining/*in situ*-hybridization-compatible tissue hydrogel) and RTF (Rapid clearing method based on Triethanolamine and Formamide)^13,14^. All of these methods clear tissue effectively; however, they are largely known to compromise endogenous fluorophore integrity^15^.

BABB was also initially found to reduce endogenous fluorophore (GFP) expression in tissue samples^16,17^. However, recent studies in brain tissue show that this can be alleviated by titration of the reagents to pH 9.5 with triethylamine and use of tert-butanol as the dehydration reagent^10,18–23^. Modified BABB clearing has yet to be embraced in skin and soft tissue in the context of wound healing and fibrosis research. We demonstrate the use of a modified BABB protocol to clear skin and soft tissue samples using a variety of transgenic mouse models. Using this protocol, we dramatically increase the depth of tissue architecture visualization and fluorophore signal captured using both fluorescent and confocal microscopy. This technology permits detailed study of dermal and epidermal biology in the context of wound healing and fibrosis.

## Results

### Modified BABB clearing preserves endogenous fluorophores in mouse tissue

**Figure 1:**
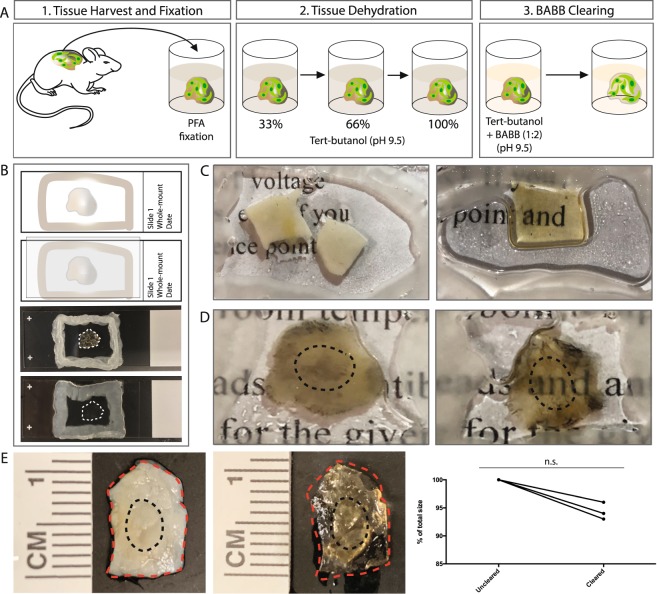
(**A**) Schematic of modified BABB tissue clearing protocol. 1. Mouse tissue harvest and fixation, 2. Tissue dehydration in progressive concentrations of tert-butanol titrated to pH = 9.5, 3. Clearing in BABB reagent (1:2 ratio of tert-butanol to BABB titrated to pH 9.5). (**B**) Schematic (top two panels – top most shows application of Vaseline® surrounding the specimen on a glass slide, panel below shows suspension of the specimen in BABB reagent and application of cover slip) with example images complementing the top two panels (bottom two panels) of whole-mounted cleared tissue specimens. Specimen edge outlined with white dotted lines. (**C**) Cleared (right panel) versus non-cleared (left panel) intact mouse skin whole-mounted in BABB reagent (right panel) versus standard mounting reagent (left panel). (**D**) Cleared (right panel) versus non-cleared (left panel) healed wounds from the dorsal surface of a mouse (harvested at post-operative day 14) whole-mounted in BABB reagent (right panel) versus standard mounting reagent (left panel). Wound scar edges outlined with black dotted lines. (**E**) Examples of cleared (middle panel) versus non-cleared (left panel) wound scar samples (harvested at post-operative day 14) demonstrating tissue shrinkage with modified BABB clearing. Graph (right panel) quantifies shrinkage of a set of specimens (n.s. = not significant, p > 0.01, paired t-test). Edge of non-cleared sample outlined with red dotted line and then overlaid on cleared specimen. Wound scar edges outlined with black dotted lines.

To evaluate the preservation of endogenous fluorophores with modified BABB tissue clearing, we prepared uninjured skin specimens harvested from L2G mice (FVB.Hsp70-luc-2A-eGFP, Jackson Laboratory). In this construct, enhanced green fluorescent protein (eGFP) expression is linked to the Hsp70A1 (heat shock protein) promotor. In uninjured tissue, eGFP expression identifies sites of increased cellular turnover/activity. These specimens were fixed using paraformaldehyde to minimize tissue autofluorescence^12^. The tissue samples were then dehydrated in tert-butanol (rather than standard ethanol or methanol) at a pH of 9.5 to optimize fluorophore preservation^10^. Our clearing protocol employed a 1:2 ratio of tert-butanol and benzoic acid:benzyl benzoate titrated to a pH of 9.5 with triethylamine (see Methods, Fig. 1A).

**Figure 2:**
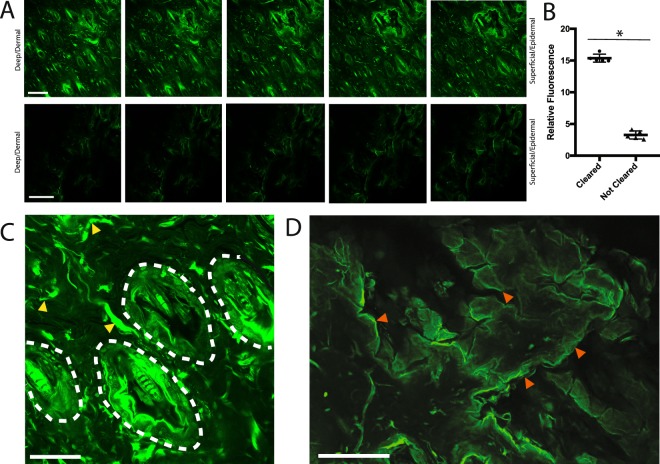
(**A**) Upper panels represent a z-stack image of cleared, uninjured L2G mouse skin (begins at left with deepest/dermal aspect, extending to most superficial/epidermal aspect at far right). Lower panels represent a z-stack image of non-cleared, uninjured L2G mouse skin from an adjacent area on the same animal as above. (Imaged on SP8 confocal microscope using the 20X objective, working distance = 680 μm with oil immersion, NA = 0.75). (**B**) Comparison of fluorescence intensity seen in cleared tissue from uninjured L2G mouse skin with comparable non-cleared tissue (*statistically significant, p < 0.0001, unpaired t-test). (**C**) Epidermal and dermal biology becomes distinctly visible with clearing. (White dotted lines highlight hair follicles, dermal fibroblasts indicated with yellow arrowheads, imaged on SP8 confocal microscope using the 40X objective, working distance = 240 μm with oil immersion, NA = 1.30). (**D**) 40X confocal imaging of cleared, uninjured L2G mouse skin whole-mounted with the hypodermis facing upwards (closest to the objective), shows that epidermis (farthest from the objective) can be clearly visualized through the full thickness of the tissue (red arrowheads highlight edges of keratinocyte “sheets” characteristic of epidermal biology, 40X objective, working distance = 240 μm with oil immersion, NA = 1.30). Experiments conducted with n = 3 biological replicates per condition (where applicable), scale bars represent 200 μm (unless otherwise indicated).

After clearing, the L2G mouse tissue was whole-mounted in BABB reagent (see Methods, Fig. 1B). Notably, the tissue was translucent after this clearing protocol (Fig. 1C,D), similar to studies using a modified BABB technique with brain tissue^22,23^. For whole mount samples, tissue clearing could be observed as early as 30–50 minutes incubation in BABB and was found to be optimal at 7 hours incubation (Table 1). Negligible tissue shrinkage (less than 7% change in tissue specimen surface area) was observed with clearing (Fig. 1E). We then imaged the tissue samples using confocal microscopy (Fig. 2). Non-cleared tissue specimens taken from the same mice at adjacent sites were used for comparison and as control.

**Table 1:** Recommended BABB incubation times for mouse skin and scar specimens.

Specimen Type	Preliminary clearing observed (incubation time)	Optimal clearing achieved (incubation time)
8 μm tissue cryosections on glass slides (uninjured skin or wound tissue)	3 minutes	30 minutes
Whole mount mouse uninjured skin specimens	30 minutes	7 hours
Whole mount mouse wound specimens	50 minutes	7 hours

Using this pH-balanced, modified BABB clearing approach, we found that the epidermal, dermal and hair follicle biology were dramatically more visible (Fig. 2A, top panels) compared with non-cleared control specimens taken from an adjacent area of skin on the same animal (Fig. 2A, bottom panels). Quantitatively, the endogenous fluorophore visibility was significantly increased in the cleared specimen (Fig. 2B). Epidermal and dermal biology were distinctly visible throughout the cleared specimen (Fig. 2C). In a cleared specimen mounted with the hypodermis facing upwards (closest to the objective), epidermis (farthest from the objective) could be clearly visualized indicating that the full thickness of the tissue could be penetrated and as such, the entirety of the whole-mount specimen could be imaged using this technique (Fig. 2D). We measured cleared uninjured L2G specimens with calipers and were able to assess that we could visualize at least 0.5 mm in tissue thickness (compared with only 4–6 μm in non-cleared tissue).

### Dermal biology revealed with BABB clearing technique

**Figure 3:**
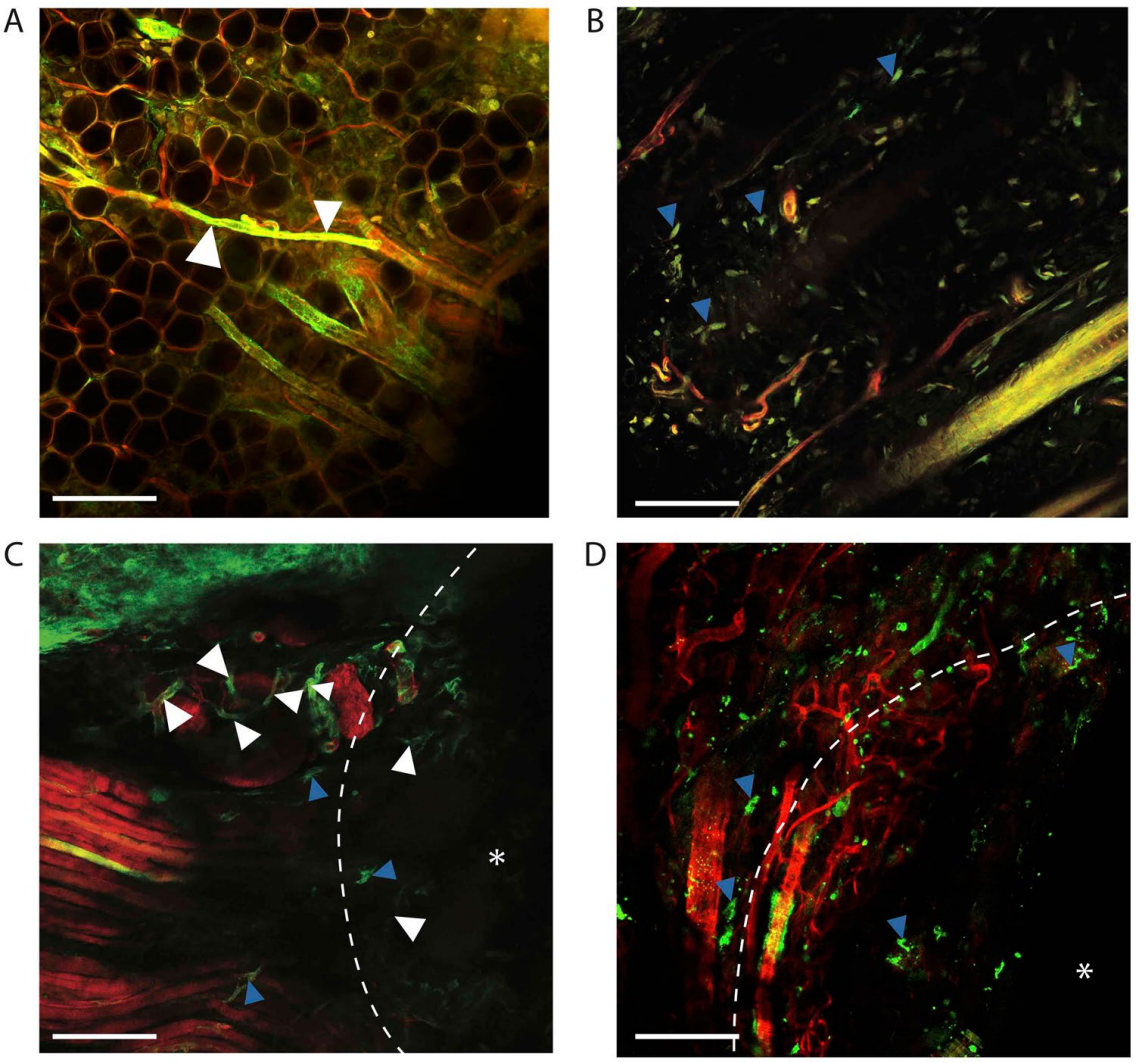
(**A**) Example of cleared, uninjured dermis from an αSMA-Cre^ERT2^::Rosa26^mTmG^ mouse. Vasculature indicated by white arrowheads. (**B**) Example of cleared, uninjured dermis from a PDGFRα-Cre^ERT2^::Rosa26^mTmG^ mouse. Blue arrowheads mark dermal fibroblasts. (**C**) Example of cleared, dermal scar tissue from an αSMA-Cre^ERT2^::Rosa26^mTmG^ mouse. Blue arrowheads indicate activated dermal fibroblasts, neovascularization associated with wound healing indicated by white arrowheads, white asterisk marks the scar center, white dotted line highlights the closing edge of the scar. (**D**) Example of cleared, dermal scar tissue from a PDGFRα-Cre^ERT2^::Rosa26^mTmG^ mouse. Blue arrowheads indicate dermal fibroblasts, white asterisk marks the scar center, white dotted line highlights the closing edge of the scar. Images taken using the 20X objective, working distance = 680 μm with oil immersion, NA = 0.75 on an SP8 confocal microscope (unless otherwise indicated). Experiments conducted with n = 3 biological replicates per condition (where applicable), scale bars represent 200 μm (unless otherwise indicated).

Looking at the dermis, we cleared tissue specimens from alpha-smooth muscle actin (αSMA)-Cre^ERT2^ mice and platelet derived growth factor receptor-alpha (PDGFRα)-Cre^ERT2^ mice bred with Rosa26^mTmG^ mice. αSMA and PDGFRα are both fibroblast markers that label fibroblasts in the dermis. αSMA is also expressed by pericytes and smooth muscle cells associated with dermal vasculature. With the mTmG construct, all cells are labelled with membrane (m)Tomato, but with Cre induction, cells expressing the Cre driver (αSMA or PDGFRα in this case), express mGFP. After clearing induced, uninjured tissue from these animals, dermal architecture could be appreciated in great detail inclusive of vasculature and adipocytes, compared with non-cleared tissue from healthy litter mates (Fig. 3A,B).

### Modified BABB clearing improves imaging of full-thickness wound healing biology

Using the aforementioned αSMA-Cre^ERT2^::Rosa26^mTmG^ mice and PDGFRα-Cre^ERT2^::Rosa26^mTmG^ mice, we created full-thickness dorsal wounds with splinting of the wound edges following an established technique which mimics human wound healing kinetics^24^. Cre-activation was pursued with local induction using liposomally-packaged 4-hydroxytamoxifen^3^. These wounds were harvested at post-operative day 14 (when mouse wound healing is complete). New vascular growth along with activated dermal fibroblasts could be clearly visualized in the αSMA tissue in the wound bed (Fig. 3C), whereas proliferating dermal fibroblasts were labelled with GFP in the PDGFRα model (Fig. 3D).

### Modified BABB clearing is efficient and effective for sectioned tissue

**Figure 4:**
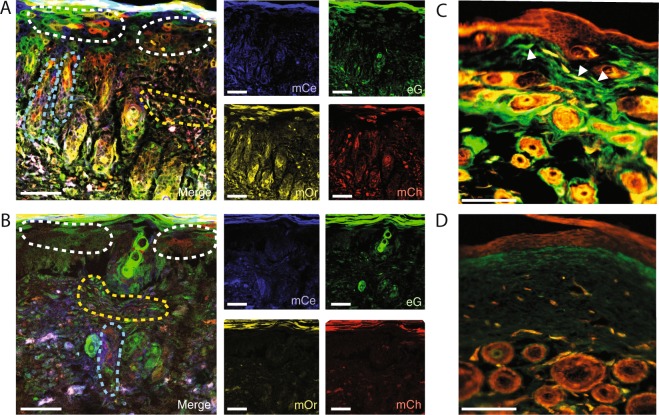
(**A**) Polyclonal expansion of keratinocytes involved in wound coverage (outlined with white dotted line), hair follicles near the edge of the wound show polyclonal proliferation (examples outlined with blue dotted lines), fibroblasts involved in wound healing (example outlined in yellow dotted line), in a cleared dorsal wound tissue section from an Actin-Cre^ERT2^::Rosa26^VT2/GT3^ mouse harvested at post-operative day 14. Merged images at left, individual channels at right (mCe = membrane (m) Cerulean, mG = mGFP, mOr = mOrange, mCh = mCherry). Images (**A**,**B**) taken at 20X using an SP8 confocal microscope (working distance = 680 μm with oil immersion, NA = 0.75), scale bars represent 200 μm. (**B**) Non-cleared tissue section equivalent to 4 A. (**C**) Cleared En1-Cre::Rosa26^mTmG^ peri-wound tissue. White arrowheads indicate En1-lineage dermal fibroblasts. Images (**C**,**D**) taken using a 20X objective using standard fluorescence microscopy (Leica DMI4000 B automated inverse fluorescence microscope), scale bars represent 150 μm. (**D**) Non-cleared En1-Cre::Rosa26^mTmG^ peri-wound tissue equivalent to 4 C. Experiments conducted with n = 3 biological replicates per condition (where applicable), scale bars represent 200μm (unless otherwise indicated).

We wondered if the modified BABB technique could be equally effectively applied to sectioned specimens from mice expressing endogenous fluorophores. We explored this with sectioned tissue from mice having undergone dorsal wound healing in Actin-Cre^ERT2^::Rosa26^VT2/GT3^ Rainbow mice. In the Rainbow mouse model, at the time of Cre-induction, all cells express one of four fluorophores (eGFP, mCherry, mOrange, mCerulean) and all daughter cells express the same color as the parent cell permitting highly accurate lineage tracing. Cre-induction was again pursued with local application of liposomal 4-hydroxytamoxifen at the time of wounding. Wounds were harvested at post-operative day 14. Sectioned specimens were exposed to the modified BABB reagent as a mounting agent. The wound sections became translucent within just a few minutes (Table 1). Clearing was optimal by 30 minutes and retained over time. In the context of wound healing, the clearing technique shows preservation of endogenous fluorophore expression while allowing extremely detailed visualization of the tissue biology (Fig. 4A) compared with non-cleared control tissue (Fig. 4B).

Finally, we explored imaging tissue sections cleared using the modified BABB technique using a standard fluorescent rather than confocal microscope. We found that clearing of Engrailed-1 (En1)-Cre::Rosa26^mTmG^ mouse wound specimens, in which fibroblasts of En-1 lineage express mGFP while other cells express mTomato, once again showed a prominent difference in the clarity and visibility of the distinct tissue biology. Specifically, individual dermal fibroblasts of the En-1 lineage were highlighted and could be individually isolated in the cleared compared with uncleared control specimens (Fig. 4C,D).

## Discussion

Tissue clearing is a valuable technique to improve tissue penetration and endogenous fluorophore visualization with microscopy. To achieve this, tissue dehydration followed by lipid solvation provides a stable refraction index throughout a tissue sample. Specifically, the dehydration process adjusts the refraction index of most proteins to be above 1.5^12^, while lipid solvation causes the tissue to become translucent. Use of a mounting media with a similar refractive index to the cleared tissue, can further optimize and maintain the clarity.

A variety of methods exist to clear tissue samples, including solvent based (e.g. BABB, 3DISCO, and Spatleholz), simple immersion (e.g. sucrose, ClearT, ClearT2, and FocusClear), hyperhydration (e.g. ScaleA2 and CUBIC), and hydrogel embedding (CLARITY, PACT, PARS)^12,13,25^. More advanced tissue clearing techniques include RTF, which requires intracardial perfusion of the mice prior to harvest^14^. Additionally many of these reagents are expensive, while the modified BABB protocol stock supplies are inexpensive (less than USD $100) and allow for clearing of hundreds of specimens. Several clearing protocols unlike the modified BABB protocol have multiple technical procedures, which are known to have a steep learning curve. For example, using CLARITY, the tissue polymerizes with hydrogel and is cleared using sodium dodecyl sulfate (SDS) with or without electrophoresis^26^. Another limitation associated with some of these techniques, for example CUBIC and ScaleA2, is that these protocols take two weeks to conduct, while the modified BABB technique can be performed in hours^27,28^.

Clearing methods have largely been found to compromise expression of endogenous fluorophores^15^, which may explain why tissue clearing is popular in neuronal studies, but less commonly applied in projects exploring other tissue types. For example, DBE, ScaleA2, and CUBIC have all been shown to specifically decrease tissue fluorophore expression^15,25^. Some of these methods also compromise tissue integrity. For example, DBE was found to shrink tissue size, while CUBIC and ScaleA2 increase tissue size^15,29^. ScaleA2 was also found to make tissue less transparent compared with other tissue clearing methods^15,28,29^. The modified BABB protocol demonstrates adequate tissue transparency and minimal tissue shrinkage as well as fluorescence preservation within the skin to allow for excellent visualization.

Although conventional BABB clearing was also initially found to compromise endogenous fluorophore intensity in tissue samples^16,17^, as well as cause significant tissue shrinkage (up to 50%)^16^, recent studies in neural tissue found that these issues can be avoided with use of tert-butanol as the dehydration reagent and titration of the reagents to pH 9.5^10,18–23^. Other studies have used tert-butanol as the dehydration reagent, but without pH balancing to 9.5^30–33^. Higher pH levels are found to increase fluorophore intensity during visualization^34,35^. The modified BABB clearing technique, coined FluorClearBABB in papers exploring it’s use in mouse brain tissue, has been used to visualize expression of virally-linked and other endogenous fluorophore models (AAV9-GFP, AAVrh10-GFP, CLIO-FITC, lectin-FITC, and Thy1-GFP) in the central nervous system, as well as to conduct retrograde mono-trans-synaptic tracing using the vGluT2-Cre transgenic mouse model^19–23^. This method is also effective at clearing heart and kidney tissue specimens while preserving fluorescent immunolabelling^36^. Additional advantages of BABB include that this approach is extremely simple, inexpensive and uses widely available organic solvents^10^. FluorClearBABB has yet to be incorporated to enhance imaging of fluorescent tissue in skin and soft tissue in the context of wound healing and fibrosis.

We successfully applied a modified BABB tissue clearing technique in the study of skin and soft tissue biology and found that it preserves expression of endogenous fluorophores allowing detailed visualization of epidermal and dermal biology at homeostasis and after injury. We observed minimal tissue shrinkage (<7%) using this protocol. We demonstrate the effectiveness of this protocol in a variety of transgenic mouse models using fluorescence and confocal microscopy to image both whole-mount and sectioned tissue using both uninjured skin as well as wound healing models. This technique can be easily reproduced by researchers studying comparable tissues in a variety of fields including regenerative medicine, cancer biology and plastic surgery.

## Methods

### Transgenic mouse models

Hsp70A1-L2G (FVB.Hsp70-Luc-2A-eGFP, Jackson Laboratory, USA), Actin-Cre^ERT2^::Rosa26^VT2/GT3^ (courtesy of Dr. Irv Weissman, Stanford University), αSMA-Cre^ERT2^ (courtesy of Dr. Ivo Kalajzic, University of Connecticut), PDGFRα-Cre^ERT2^ (Jackson Laboratory, USA) and Rosa26^mTmG^ (Jackson Laboratory, USA) mice were used in this study. Cre-^ERT2^-inducible mice were intraperitoneally induced with 200 μL of 20 mg/mL of tamoxifen (Sigma Aldrich) diluted in corn oil (Sigma Aldrich) continuously for 5 days or locally induced using 4-hydroxytamoxifen liposomes for 3 days^3^. All animal experiments were carried out under the guidance and approval of the Stanford University’s Administrative Panel on Laboratory Animal Care.

### Tissue preparation

All tissue specimens were fixed in 2–4% paraformaldehyde (PFA, Electron Microscopy Sciences) at 4 °C for 12–24 hours. After fixation, all samples were washed in 1X phosphate-buffered saline (PBS, Gibco) for 30 minutes x3. For dehydration, tert-butanol (FisherSci) buffered to a pH of 9.5 with triethylamine (FisherSci) was prepared with gradients of 33%, 66%, and 100% by dilution with MilliQ water. Fixed tissue specimens were placed into increasing gradients of tert-butanol (33%, 66%, and 100%) at room temperature for 30 minutes each and then left in 100% tert-butanol overnight (12–16 hours).

### Tissue clearing with BABB

Tert-butanol and benzoic acid:benzyl benzoate (Sigma Aldrich) at a 1:2 ratio were titrated to a pH of 9.5 with triethylamine. Tissue samples were placed in glassware with the prepared BABB solution for clearing for 7 hours at room temperature (Table 1). Cleared tissue can be stably stored in this solution at 4 °C or whole-mounted for immediate imaging.

### Whole-mounting tissue samples

A rectangular border of Vaseline was prepared in the middle of a Superfrost/Plus microscope slide and the center of the rectangle was filled with 500 μL of mounting medium (BABB or fluoromount-G (SouthernBiotech)). The tissue sample was placed into the reservoir of mounting medium and a cover slip was applied carefully to avoid air bubbles overlying the specimen. The whole-mounted samples were stored at 4 °C.

### Preparation of sectioned tissue

For sectioned specimens, tissue samples were maintained in 30% sucrose in 1X PBS at 4 °C for at least 48 hours after fixation. The samples were then incubated in O.C.T. (Tissue Tek) for at least 24 hours at 4 °C, followed by embedding in O.C.T. and freezing in tissue molds on dry ice and stored at −20 °C. The frozen blocks were mounted on a MicroM HM550 cryostat, sectioned at 8 μm thick and transferred to a superfrost/Plus adhesive slide. 50–100 μL of prepared BABB solution (pH = 9.5) was then applied directly to the tissue sections for clearing. A cover slip was placed over and the edges of the slip sealed to the slide with clear nail polish. The slides were stored at 4 °C for 30 minutes prior to imaging.

### Fluorescence and confocal imaging

Fluorescent microscopy was conducted using a Leica DMI4000 B automated inverse fluorescence microscope. Confocal microscopy was conducted using a Leica WLL TCS SP8 Confocal Laser Scanning Microscope (Stanford Cell Sciences Imaging Facility, purchased with NIH grant 1510OD01058001A1). For confocal imaging, the working distance and numerical aperature (NA) for the objectives used are as follows: 20X (680 m with oil immersion, NA = 0.75) and 40X  (240 μm with oil immersion, NA = 1.30). Confocal imaging was conducted with a 2048 × 2048 pixel format and 200 Hz speed (as noted above). Z-stack step size was 0.3 μm. Precise excitation and hybrid detection of the fluorophores found in each mouse model used were captured. Z-stack images were rendered into 3-dimensions for analysis.

### Fluorescence quantification and data analysis

ImageJ (Fiji, National Institutes of Health) software was used for analysis of imaging data. Raw image stacks were imported, two-dimensional confocal micrographs were generated and fluorophore expression intensity was quantified. Images presented in the manuscript are represented as the average maximal projection of 8 μm tissue sections with brightness and contrast adjusted accordingly for each color channel. Quantitative analysis was conducted using GraphPad Prism 6.

All methods were carried out in accordance with relevant guidelines and regulations. All experimental protocols were approved by Stanford University.

## Data Availability

Data generated or analyzed during this study are included in this published article.
